# A Demonstration of ‘Broken’ Visual Space

**DOI:** 10.1371/journal.pone.0033782

**Published:** 2012-03-29

**Authors:** Ellen Svarverud, Stuart Gilson, Andrew Glennerster

**Affiliations:** 1 Department of Optometry and Visual Science, Buskerud University College, Kongsberg, Norway; 2 Department of Physiology, Anatomy and Genetics, University of Oxford, Oxford, United Kingdom; 3 School of Psychology and Clinical Language Sciences, University of Reading, Earley Gate, Reading, United Kingdom; University of Muenster, Germany

## Abstract

It has long been assumed that there is a distorted mapping between real and ‘perceived’ space, based on demonstrations of systematic errors in judgements of slant, curvature, direction and separation. Here, we have applied a direct test to the notion of a coherent visual space. In an immersive virtual environment, participants judged the relative distance of two squares displayed in separate intervals. On some trials, the virtual scene expanded by a factor of four between intervals although, in line with recent results, participants did not report any noticeable change in the scene. We found that there was no consistent depth ordering of objects that can explain the distance matches participants made in this environment (e.g. A>B>D yet also A<C<D) and hence no single one-to-one mapping between participants' perceived space and any real 3D environment. Instead, factors that affect pairwise comparisons of distances dictate participants' performance. These data contradict, more directly than previous experiments, the idea that the visual system builds and uses a coherent internal 3D representation of a scene.

## Introduction

**Figure 1:**
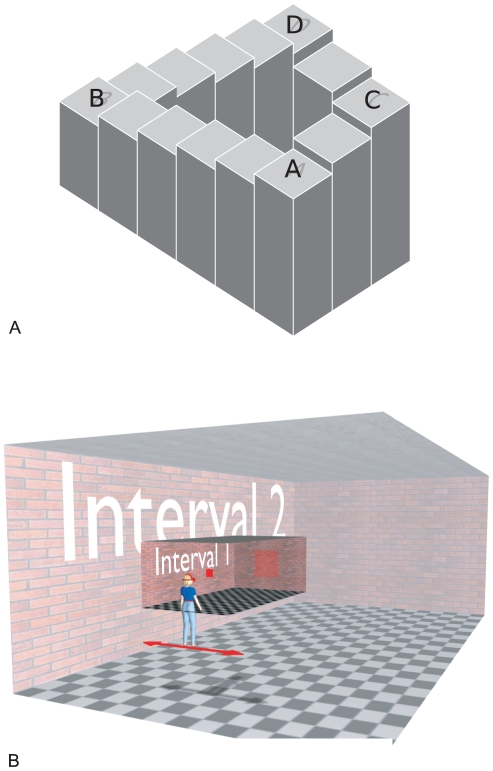
Logic and setup of the experiment. A: ‘Penrose stairs’ illusion. In the real world, a continuously ascending or descending staircase like this would be impossible. Is step A above or below step D? A similar paradox emerges in our experiment in relation to the perceived distance of objects in an expanding room. B: Virtual scene. The high and low contrast regions illustrate the scene in intervals 1 and 2 of a trial in which the room expanded. Participants moved from side to side to generate motion parallax and compared the perceived distance of two squares, one presented in each interval. Shadows and arrow are for illustration only.

Artists such as Escher [1] have often exploited paradoxes that emerge when a 3D scene is depicted by means of a flat, 2D picture. In Figure 1A, for example, point A in the image can been seen to be above point D if you follow the stairs via B and yet below point D if you follow a route via C. This failure of transitivity (A>B>D and yet A<C<D) is possible in a drawing but there is no physically realisable 3D structure that would show the same properties: in the real world, relationships such as ‘above’ or ‘farther than’ are transitive. The illusion is possible because drawings of 3D scenes are inherently ambiguous, with each point on the picture plane defining a visual direction but not a distance, so there is no one-to-one relationship between the picture and 3D locations in space.

The same is not true of an actual 3D representation or model. Most theories of 3D vision and spatial representation assume that humans generate a 3D representation of space, i.e. one with an origin and three axes, and it is usually assumed that this is constructed first in an ego-centric coordinate frame and then in a world-based frame [2], [3], [4]. It is often argued that the visual representation may be distorted [5], [6], [7], [8], [9], [10], but with a one-to-one mapping between points in the internal representation and those in the external world. However, there has been a debate about whether the notion of an internal representation, or visual space, is necessary [11], [12] and whether it can be sustained in the face of recent evidence [13], [14].

In order to test this model, we used a paradigm in which participants fail to notice anything unusual when the scene around them expands or contracts by as much as fourfold (i.e. a 16-fold range in scale overall), viewed in immersive virtual reality [15], [16], [17]. This astonishing lack of awareness of object size and distance is potentially highly informative about the central processing of spatial information, in the same way that knowing the set of stimuli that are treated as equivalent inputs to a cell informs neurophysiologists about the operations it carries out [18]. For further discussion of the expanding room phenomenon, see [15], [16], [17]. Briefly, participants report that they do not notice anything odd about a room that expands or contracts. Additionally, in other similar experiments, participants' behaviour suggests that they are unable to separate trials in which the room expands from those in which it contracts [16].

In our experiment, we tested whether a one-to-one mapping between an internal representation and the external scene could explain performance on judgements of object distance. In Figure 1A, there is no consistent way to determine whether ‘A’ is above or below ‘D’; in our experiment, we tested whether participants perceived one object (‘A’) to be in front of or behind another object (‘D’) when tested via two separate intermediates (‘B’ and ‘C’).

## Results

Figure 1B shows the virtual environment used in the experiment. Participants wore a wide field of view, high resolution head mounted display tracked with six degrees of freedom with low latency and high spatial precision using an optical tracking system (see Materials and Methods) so that participants had a fully immersive experience of a simulated 3D environment. The virtual scene was a brick-textured room with a chequered floor, as shown. Participants viewed a square in interval 1 and compared its distance to that of a similar square displayed in interval 2 while rocking from side to side to enhance the motion parallax information. At the start of each interval, the squares were always the same angular size (5.7 deg), so this was not a useful cue to distance. The distance of the square in interval 1 was fixed for each condition and always displayed at eye height. Participants responded by pressing one of two buttons to indicate whether they perceived the square in interval 2 to be nearer or farther away than the reference square in interval 1. The distance of the square in interval 2 was varied from trial to trial according to a staircase procedure (see Materials and Methods) to establish the distance at which participants perceived the two squares to be at the same distance.

On half of the trials, the room changed size between intervals by a factor of four, as illustrated in Figure 1B. When the room was small (2.35×4.50×1.55 m) the floor came to about waist height, while when it was large (9.4×18×6.2 m) the gap between the participant's feet and the floor was as high as they were tall. But the participants could not see their own body. The texture of the room was scaled with the room so that there was the same number of bricks on the walls and tiles on the floor and ceiling in both room sizes. Since the room was visible throughout the trial, an important feature of the expansion was that the change occurred without any perceptible visual signal. Subjectively, the transition was seamless. In none of the trials, neither those on which the room expanded nor those on which it remained static did participants notice any change in the size of the room [15], [17]. This is consistent with previous findings using large-scale stimuli in which looming cues are eliminated [19], [20]. However, despite the subjective perception of a stable room, there is evidence that participants remain sensitive to the true distance of objects and weight this information to a greater extent when the target is close to the viewer or to other visible references [17].

**Figure 2:**
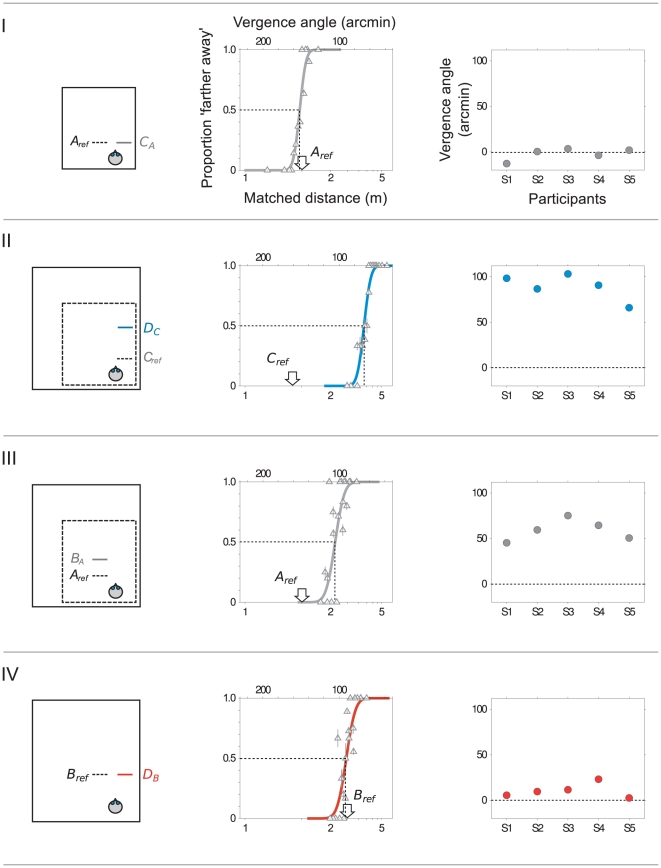
Four interleaved distance comparisons. Plan views (left) show how the room remained the same size between intervals (Rows I and IV) or expanded (Rows II and III), not drawn to scale. In each case, the position of the reference square in interval 1 is shown by the dashed line and the comparison square (interval 2) by a solid line. The psychometric functions show the proportion of trials on which the comparison square was judged to be ‘farther away’ than the reference. The arrows show the distance to the reference square (in arc minutes and metres on top and bottom axes, respectively) and the dashed line shows the point of subjective equality (PSE). Plots in the right hand column show participants' biases, i.e. the difference between the reference and the PSE (expressed in arcmin). In most cases, standard error of the PSEs, obtained from the probit fit, are smaller than the size of the markers. Although not shown here, square B and C were each presented at two reference distances (*B_ref1_*, *B_ref2_*, *C_ref1_*, and *C_ref2_*). The reference distances illustrated here are *B_ref1_* and *C_ref1_*. Similarly, the biases for square D shown in red and blue are those obtained with references *B_ref1_* and *C_ref1_*, namely PSE *D_B1_* and *D_C1_* (see text for details).

Figure 2 shows the four conditions we used in our experiment (first column). For the actual location of the reference squares, see Procedures S1. The data shown in column 2 are from a single run of 400 trials, 100 trials per condition, randomly interleaved during the run but analysed separately. The first row illustrates a condition in which the room remained a constant size (in this case, small) between interval 1 and 2. Participants had to match the distance of square A in the middle of the room with square C which was placed closer to the right hand wall. As one might expect, given that the room remained a constant size during the trial, participants were able to do this quite accurately. The psychometric function in the centre shows the proportion of trials on which this participant (S1) perceived the comparison square, C, to be farther away than the reference, A. The data are fitted by a cumulative Gaussian function whose mean indicates the distance at which the reference and comparison squares were perceived to be equidistant (point of subjective equality, or PSE) shown by the dashed line. Table 1 shows the conventions used in the paper for labelling reference distances and points of subjective equality: in this case, PSE *C_A_* is very similar to the reference distance, *A_ref_*. The third column shows that the same is true for other participants, i.e. the bias is small (−2.20±6.54 arcmin, mean ± s.d.). This is equivalent to a bias of about 1 cm at a reference distance of 75 cm, as in this case.

**Table 1:** Labels used for reference distances and points of subjective equality (PSEs).

Room expansion	Reference distance	Distance of PSE
Expanding	*A_ref_*	*B_A_*
Static (large)	*B_ref1_* or *B_ref2_*	*D_B1_* or *D_B2_*
Static (small)	*A_ref_*	*C_A_*
Expanding	*C_ref1_* or *C_ref2_*	*D_C1_* or *D_C2_*

Runs were repeated using two different reference distances for square B (i.e. *B_ref1_* or *B_ref2_*) and similarly two values for square C. For points of subjective quality, subscripts indicate the reference square: e.g. *B_A_* refers to the PSE when square B, shown in interval 2, appeared to be at the same distance as square A, shown in interval 1.

The second row of Figure 2 shows results when the room expanded between intervals and the reference square, C, was close to the wall. (The location of the reference square C (and B) varied slightly between runs, as explained below. Values of the reference distances are shown in Figure S1.) In this case, with the reference close to the wall, there is a large bias caused by the room expansion. For example, a bias of 80 arcmin corresponds to a comparison square that is at a distance 174 cm farther away than the reference square. The third row of Figure 2 shows results for the condition in which the reference square was placed away from the wall (square A), as was the comparison square (B). Again, the room expanded between intervals and here, too, there was a bias in distance judgements but in this case the bias was significantly smaller (Row II: mean bias 88.9±14.3 arcmin; Row III, mean bias 59.0±11.7 arcmin; p<0.0001, using a bootstrap method) [21]. This difference is compatible with previous results showing larger biases in distance matching when the target is close to visible references [17]. The importance of proximity between the target and the surroundings for the ‘texture-based’ cue, a catch-all term here for any cue that indicates the distance to the square in relation to the room rather than its physical distance, is easy to understand. If other objects were infinitely far away the only cues left would be ‘physical’ ones such as vergence. When the target is close to the wall, however, cues such as relative disparity indicate its location relative to a point on the wall and hence, for example, its distance as a proportion of the distance to the back wall. Finally, the fourth row of Figure 2 shows biases when the room is stable throughout the trial (like the first row) but this time with the room enlarged. As expected, the biases are again relatively small (mean 10.5±7.89 arcmin).

These differing biases suggest that it may be difficult to pin down the location of squares A, B, C and D in a single coherent frame. Participants believed themselves to be in the same room throughout the experiment and never perceived the room to change in size. Yet, the data in Figure 2 suggest that there may be no consistent representation of location in which depth ordering of pairs of objects can be preserved. This is because the route from reference square A to comparison square D via square B in the centre of the room (i.e. Rows III and IV in Figure 2) involves smaller biases than a similar comparison of square A and D via comparison square C near the wall (Rows I and II of Figure 2). However, to test this impression rigorously, some care is required. Theoretically, one would like to compare two conditions under which the perceived distance of the reference square A in Figure 2 is the same as the perceived distance of the square D and to do so via two separate routes (shown in Rows I+II or III+IV). Specifically, the ideal comparison would be:
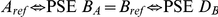
and
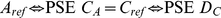
(1)where *A_ref_*, *B_ref_* and *C_ref_* are the distances of reference square A, B and C respectively, PSE *B_A_* is the distance of square B at the point of subjective equality relative to reference square A, etc, and 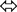 means ‘are at an equal perceived distance’. The ‘ = ’ sign means ‘is identical to’ because it equates the distance of a square, e.g. B, under identical conditions (size of room, location in room). For example, if square B was placed at the point of subjective equality relative to square A (i.e. at PSE *B_A_*), it would be an identical stimulus to reference square B placed at the same distance (PSE *B_A_* = *B_ref_*).

Of course, when running all the experiments together, it is impossible to know in advance the value of PSE *C_A_* and PSE *B_A_* (since these depend on the participant's responses during the experiment). This means it is not possible to arrange for the reference squares, shown at distances *B_ref1_* and *C_ref1_*, to be exactly equal to PSE *B_A_* and PSE *C_A_*, respectively, as we would like. Instead, in pilot experiments, we found approximate values for *B_ref_* and *C_ref_* for each participant and then ran the main experiment twice over using two different reference distances, with the aim of having one closer and one more distant than the expected ‘ideal’ reference value. This was almost always achieved, as the pilot generally provided a good estimate of the ‘ideal’ reference distance in the experiment. On the rare occasions it was not, one of the references was usually very close to the ideal reference distance (see Procedures S2 and Figure S1).

**Figure 3:**
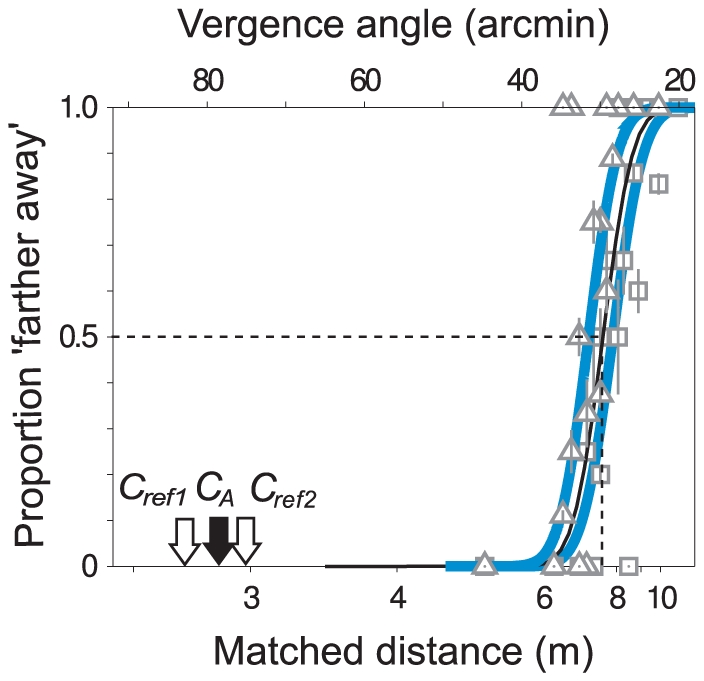
Inferring points of subjective equality (PSEs). Because we ran all the conditions simultaneously, the appropriate distance for the reference squares B and C could not be determined precisely in advance. Instead, two reference distances close to the expected value were chosen and interpolation (or, rarely, extrapolation) used to estimate the PSE that would have been obtained had the reference been positioned at the ‘ideal’ distance (*C_A_*, black arrow). Two reference locations (*C_ref1_* and *C_ref2_*, open arrows) and the corresponding psychometric functions are shown, together with the interpolated curve (black) and inferred PSE (dashed line). See also Figure S1.

Figure 3 shows how data using these two reference distances can be used to estimate the distance at which square D would be perceived to be equidistant with a square at the ‘ideal’ reference distance (in this case, PSE *C_A_*). The data shown were collected in two separate runs as described above, with the reference square C at distance *C_ref1_* in one run and at *C_ref2_* in another. For the more distant reference, the psychometric function was shifted to a farther distance, as expected. The distance of the ‘ideal’ reference, PSE *C_A_*, is shown by the black arrow (PSE *C_A_* is known at the end of the experiment but not in advance). By design, the two reference distances, *C_ref1_* and *C_ref2_*, span the location of this hypothetical ‘ideal’ reference. Linear interpolation can be used to recover the expected PSE assuming that the reference had been at *C_A_*, as illustrated by the thin black curve lying between the blue psychometric curves in Figure 3. In this way, we derived the expected PSEs for all conditions, i.e., the distances at which square D was perceived to be at the same distance as square A, either via intermediate square B or intermediate square C. The original PSEs (e.g. for references at *C_ref1_* and *C_ref2_*) are shown in Figure S1.

**Figure 4:**
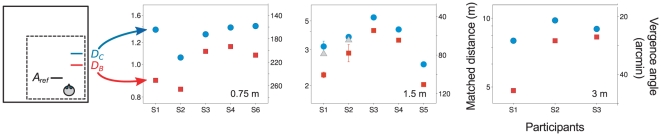
Dual perceived distances of an object. Red squares show the interpolated PSEs at which the comparison square D was perceived to be at the same distance as the reference square A when this was judged via an intermediate square B (shown as perceived distance or corresponding vergence angle). Blue circles show the equivalent PSEs when the intermediate object was square C. Data are shown for five participants when reference square A was at 0.75 m and 1.5 m and for three participants at 3 m. Error bars showing standard deviations are shown for four points at 1.5 m (see Procedures S2). For two participants (S1 and S2), PSEs obtained for a direct comparison between reference square A and comparison square D are shown as the grey triangles in the middle panel (see Procedures S2). The PSEs used for the interpolated values presented here are shown in Figure S2.

Figure 4 shows the derived PSEs when the distance to reference square A was 0.75, 1.5 and 3 m. Red squares show the point of subjective equality for square D when the intervening comparison was via square B (PSE *D_B_*, i.e. the conditions illustrated in Rows III and IV of Figure 2), while blue circles show the equivalent PSE when the intervening distance judgement was with square C (PSE *D_C_*, see Rows I and II of Figure 2). In every case, the distance of square D that was perceived to be the same as the distance of square A was greater when the judgement was made via the intermediate square C than via square B. Even applying a simple sign test [22], if we assume each of the runs shown in Figure 4 is an independent test of the null hypothesis, the difference between conditions is highly significant (N = 13, p = 0.0003). The difference between the two routes (i.e. PSE *D_B_* and PSE *D_C_*) can also be tested in a way that takes account of the variability across individuals using a bootstrap method and is again significant for all three distances (0.75 m, p<0.0001; 1.5 m, p<0.0001; 3 m, p = 0.008).

**Figure 5:**
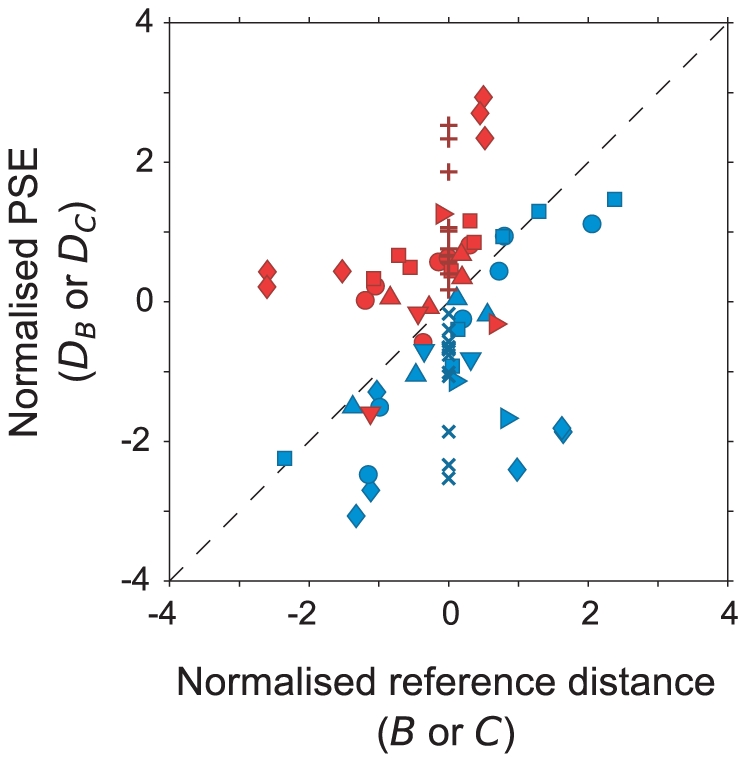
Normalised values of PSE for square D plotted against normalised values of the reference distance. Zero on the abscissa (*x*_0_) is the vergence angle at the ‘ideal’ reference distance, i.e. the PSE *B_A_* or *C_A_*. The difference between this ‘ideal’ value and the vergence angles of the reference squares (presented at distances *B_ref1_*, *B_ref2_*, *C_ref1_* or *C_ref2_*) was divided by the standard deviation of the psychometric function that gave rise to PSE *B_A_* or *C_A_* (*σ_x_*), so that, in effect, the reference distances are plotted as z-scores (*x* = (*x*_1_−*x*_0_)/*σ_x_*, where *x*_1_ is the vergence angle of the reference surface and *x* is the value plotted; see Procedures S2 for details). Similarly, zero on the ordinate is the expected PSE for D if the reference was at the ‘ideal’ distance (the mean of PSE *D_B_* and PSE *D_C_*, expressed as a vergence angle, *y*_0_). The difference between this ‘ideal’ PSE and the actual PSEs measured (*D_B1_*, *D_B2_*, *D_C1_* and *D_C2_*, expressed as a vergence angles, *y*_1_) were divided by the root mean square standard deviation of the psychometric functions (*σ_y_*) that gave rise to PSE (*y* = (*y*_1_ *−y*_0_)/*σ_y_*; see Procedures S2 for details). As in Figure 4, red symbols show data for route A – B – D and blue symbols for the route A – C – D. Different symbols shapes are used for different participants. The red plusses and blue crosses re-plot the interpolated data from Figure 4 on these relative axes. They are shown at a reference vergence angle of zero, by definition in this plot, since the notional reference is always the ‘ideal’ reference distance (PSE *B_A_* or *C_A_*).

The statistics quoted above do not rely on any estimate of the precision with which individual PSEs were determined. Nevertheless, Figure 4 shows, for two participants at 1.5 m viewing distance, an estimate of the standard deviation of the PSE values; given that these are interpolated points, estimating the variability requires certain assumptions to be made (see Procedures S2). Figure 5 demonstrates a method by which the two routes (via square B or square C) can be compared without relying on interpolation/extrapolation. It uses the same raw data as Figure 4 and it supports the same conclusion but it has the advantage that the data are more directly related to the measured points of subjective equality and there is no need to calculate the PSE value for any ‘ideal’ reference distance.

Values in Figure 5 are calculated as follows. The abscissa shows the disparity of the reference (e.g. *B_ref1_* or *B_ref2_*) relative to the ‘ideal’ reference distance (in this case PSE *B_A_*). In Figure S3, these raw values are shown but, since the range of values varies with viewing distance, we have normalised them by dividing each by the standard deviation of the psychometric function that gave rise to the PSE. The ordinate shows the PSE for the match with square D (i.e. *D_B1_*, *D_B2_*, *D_C1_* or *D_C2_*), again plotted relative to the expected value which, in this case, we have taken as the mean of the PSEs measured via the two routes (i.e. mean of the two interpolated values shown in Figure 4, *D_B_* and *D_C_*). As before, these raw values are shown in Figure S3, but here we have normalised the values by an average of the standard deviation of the relevant psychometric functions (whose means are *D_B1_*, *D_B2_*, *D_C1_* and *D_C2_*; see Procedures S2 for details). Other things being equal, one would expect that the distance of the PSE should reflect the distance of the reference square so the data should lie on the diagonal of Figure 5. Any difference between the conditions (i.e. the route via B or C, red and blue symbols respectively) would result in a systematic deviation from the diagonal, as is clearly the case (t-test comparing normalised *D_B_* minus normalised distance of reference square B with normalised *D_C_* minus normalised distance of reference square C: t_50_ = 6.9, p<0.0001 and by bootstrap p<0.0001). The interpolated PSEs from Figure 4 are shown in Figure 5 as crosses/plusses, plotted at the ‘ideal’ reference distance (zero on this axis, by definition).

It has been noted earlier that previous distance matching results in an expanding room measured near-to and far-away from a wall can explain the direction of the effect we observer here. In fact, using the best fitting estimates from Svarverud et al. [17] which estimate the weight applied to ‘texture-based’ and ‘physical’ cues across different conditions, we can also predict the magnitude of the effect we would expect to see in the current experiment. The mean ratio of vergence angles to square D via C or via B is 0.77 (s.d. 0.07). Using estimates of texture-based weights, *k*, from Svarverud et al. [17] for the middle of the room and close to the wall (*k* = 0.08 and 0.42 respectively), the prediction for this ratio is 0.73.

## Discussion

If participants generated a 3D model of the scene that they observed, and used this model as the basis for their judgements, they would not make the distance matches that we have found in our experiment. The assumption that there is an internal ‘visual space’, albeit distorted compared to the real scene, does not allow for a one-to-many mapping between internal and external coordinates, nor for the intransitivity of distances we have shown here.

Of course, it is impossible to recreate the conditions we have investigated in a normal environment without virtual reality, but the conclusions we draw are based on participants' perceptions. There is no reason to suppose that the nature of the representation or the computations that underlie performance are fundamentally different in an expanding room compared to those in a stable room. Certainly, the subjective experience of the participants gives no indication that this is the case. The intransitivity we have demonstrated applies to the representation of the scene, not to the stimuli we used. If the static room and the expanding room are equivalent stimuli, in the sense of the ‘null test’ discussed earlier [18], then the conclusions we have drawn about the nature of the representation are equally applicable to a static or expanding room, even though they can only be measured in the latter case. Some have argued that any conclusion drawn on the basis of evidence gathered in virtual reality must be suspect [23], [24]. Our experience has been that results gathered in simulated static scenes have been similar to that expected in a normal scene and we have included such simulated static scenes in our experiments as a control [15], [16], [17].

The critical difference between the two types of model we have considered is whether the distance of an object can be determined purely from the information present at the time the judgement is made. According to the 3D reconstruction model discussed in the Introduction, this process is carried out once for each object and then the two distance estimates are compared. The cue combination model [15], [16], [17] instead uses a weighted combination of ‘texture-based’ and ‘physical’ cues. The ‘texture-based’ cue remain the same independent of the physical size of the room (as it indicates distance relative to the size of the room) while ‘physical cues’ such as vergence and distance walked reflect the true distance of objects. The texture-based cue does not contribute to a 3D reconstruction model because it has no meaning if the observer estimates the distance of one object at a time; it is only useful in predicting the relationship between two distance estimates. A cue combination model based on these two cues can explain our data, in the sense that it predicts larger biases for the route A – C – D than for the route A – B – D due to the greater effect of the wall in the former case, as discussed earlier. The fact that the cue combination model successfully accounts for our data suggests that pairwise comparisons may form a fundamental component of human spatial representation [25].

Intransitivity has been demonstrated in at least one other domain [26] but not, to our knowledge, for 3D perceptual space. Smeets et al. [27] have shown that, in the presence of illusions such as the Judd or Poggendorff illusion, the order in which participants make judgements matters. Although their example did not test intransitivity of a single relationship, their results were incompatible with perceptual space being an affine or even projective transformation of real space (2D space, in their case). Instead, they suggest that illusions affect single attributes without affecting others and that visual space might not exist at all. Koenderink and colleagues [13], [28] have raised the possibility that there is no single internal representation of space, in response to the discovery that changing the participant's task can radically change the distortion of visual space. For example, they found that the curvature of visual space had opposite signs depending on whether participants, who were in an open field, had to bisect two points [13] or direct a remote pointer to point towards another target [28]. They discuss the idea that the notion of ‘apparent fronto-parallel’ (i.e. flat, neither curved towards nor away from the observer) may be incoherent in the following sense. A point could be seen as lying on the fronto-parallel between two other points as measured by one task but not by a second task. Such a result, if found, “would kill the very notion of visual space” [28]. However, they did not, at the time, find the evidence conclusive.

There are many psychophysical results that are compatible with the suggestion of there being no coherent visual space even if these do not provide a critical test. For example, He et al. [29] showed that observers underestimated distance when an obstacle obscured a significant portion of the ground surface between the observer and the target but this effect disappeared when observers were asked to plan a path around the obstacle, provided the ground could be seen for the whole route. This fits with the idea that the target distance is computed ‘on-the-fly’ once the task has been set, rather than being represented explicitly as part of a 3D reconstruction. Commenting on these findings, Wu et al. [30] note that the task-dependent hypothesis they favour predicts that, contrary to everyday experience, “our space perception changes when we look around”.

Our findings provide a much stronger test of the coherence of visual space. By using a single task, by ensuring that the perceived stimulus distance was equivalent for the critical conditions and by ensuring that distance cues were the same for both ‘routes’ when participants viewed square A and D, we have been able to show that participants could not be referring to a single representation of the room, with consistent coordinates for each object, even though the room appears to them to be stable throughout the experiment [16], [17].

If the visual system does not generate a single internal 3D model of the scene from which all responses are drawn, there must be an alternative form of representation that observers use when carrying out the task. As yet, there are few detailed hypotheses about the form that such a representation might take. One suggestion has been that ego-centric, gaze-centered representation is important, with some evidence that transfer of information from previous fixations to the current gaze-centered frame results in biases that can explain human performance in pointing tasks [31]. Although it would be difficult to explain our current results in terms of gaze-centred biases, the notion of an ego-centric representation of visual direction that survives changes in gaze, albeit with some errors, is an important one. If such a representation also contains information about approximate viewing distance, it could perform many of the functions traditionally associated with an allocentric representation [25]. A representation of this type could act as a sufficiently ‘loose’ description of object location (or of raw data from which location-related properties could be computed ‘on-the-fly’) to permit many task-dependent effects to co-exist without any explicit contradiction being revealed in the representation.

One distinct alternative to 3D reconstruction is view-based representation, particularly in the contexts of object recognition [32] or navigation [33], [34], [35], [36]. However, view graphs and similar view-based representations do not represent information about the scene structure in a form that would readily allow the observer to judge whether one target is nearer or farther than another, as participants did in our experiment. A challenge for the future will be to implement representations that are less ‘rigid’ and internally consistent than a full Cartesian model and yet are sufficiently robust to allow precise and accurate control of movement. Such representations are likely to be of considerable interest in the field of robotics in applications such as simultaneous localisation and mapping [37].

## Materials and Methods

### Participants

Six participants (age 21 to 39), including one author (S1) and five unaware of the purpose of the experiment had normal or corrected-to-normal vision (6/6 or better) and normal stereopsis (TNO 60 arcsec or better). One participant (S2) had previously taken part in a different experiment using an expanding virtual room. Observers gave written informed consent to participate in this study, which was approved by the University of Reading Research Ethics Committee.

### Equipment

The virtual reality stimuli were presented on a Datavisor 80 (nVision Industries Inc, Gaithersburg, Maryland, USA) head mounted display (HMD) unit that presented separate 1280×1024 pixel images (interlaced) to each eye using CRT displays. Each eye's image was 73 deg horizontally by 69 deg vertically with a binocular overlap of 38 deg giving a total horizontal field of view of 108 deg (horizontal pixel size 3.4 arcmin). The display was fixed at an accommodative distance of 0.5 dioptres (2 m). The location and pose of the head was tracked using a seven-camera, MX3 Vicon real time optical tracker (Vicon Motion Systems Ltd, Oxford, UK) which recorded the position of individual infra-red reflective markers rigidly attached to the headset and delivered an estimate of the position and orientation (nominal accuracy ±0.1 mm and 0.15 deg, respectively) of the headset, polled at 60 Hz. This information was then used to render images for the appropriate optic centre location and display frustum of each eye's display [38]. A dual processor workstation with dual graphic cards rendered the images at 60 Hz, which were sent both eyes' displays in the HMD and, simultaneously, to the operator's display console, with a total latency from head movement to image change of approximately 34 ms.

### Stimulus and task

The participant was surrounded by a virtual room with brick textured wall, black and white checker board floor and grey ceiling tiles (see Figure 1B). The task was to judge whether a comparison square in the second interval was closer or farther away than a reference square displayed in the first interval. There were four interleaved conditions in each run, as illustrated in Figure 2, in which the virtual room either expanded between interval 1 and 2 of the trial or remained static throughout the trial. Participants did not report a perceived change in the size of the room and were not told that this might happen. They were told that the square in the first interval would be presented at different distances and locations and were instructed to turn to look directly at the square in each interval while moving from side to side to generate motion parallax information (amplitude of about 0.65 m and frequency of 0.4–0.5 Hz [17]). Participants were not given any instructions as to how they were to judge distance, e.g. physical distance or the distance relative to the room [17] but simply to judge which square appeared closer. Each run began with the participant in a virtual wireframe room, similar in size to the real room in which the experiment was carried out (about 3×3×3 m). Both the reference and comparison squares were red and displayed at eye height. Their distance was fixed relative to a point at the centre of a ‘viewing zone’ in which the participant moved laterally, to and fro, to obtain motion parallax information and, if viewed from this point, they had a constant angular size (5.7 deg) (see Procedures S1). The reference square was set at a predetermined distance for each of the four interleaved experimental conditions while the distance to the comparison square varied (see below).

In two conditions, the virtual room remained the same size in both intervals, either 2.35×4.50×1.55 m (‘small’) or four times larger in all dimensions (‘large’, 9.4×18×6.2 m (width×depth×height)). In the other two conditions, the room expanded by four times in all dimensions between the two intervals (i.e. from ‘small’ to ‘large’). The texture of the room was scaled with the room so that, for example, there was the same number of bricks on the walls and tiles on the floor and ceiling in both intervals. When the room expanded between intervals, which occurred as a linear ramp over a period of 1.0 s, it did so in such a way that there was no information about the scale change as viewed from the cyclopean point (i.e. a point half way between the left and right eyes). Although the same was not quite true of the view from the left and right eye's view points, which would have changed slightly if the participant had remained static, in practice these image changes were very small and generally masked by the larger image changes caused by the observer moving. Since the room was visible throughout the trial, an important feature of the expansion was that the change occurred without any perceptible visual signal. Subjectively, the transition was seamless.

The location of reference square A was fixed throughout any given run (at 0.75, 1.5 or 3 m) but reference distances *B_ref1_*, *B_ref2_*, *C_ref1_* and *C_ref2_* depended on the participant's responses during pilot trials. The actual location of the reference squares used in the four interleaved conditions is given in Figure S1.

A white vertical line extending from the floor to the ceiling, close to the wall and at a distance approximately equal to *A_ref_* in the small room and at a distance four times farther away when the room was large, provided a strong relative distance cue. Although this cue was useful within a trial, a random jitter in depth between trials by ±7% of the distance to reference square meant that it could not be used across trials as a reliable reference.

### Psychometric procedure

In one run of trials, there were three reference squares presented at pre-determined distances, e.g. *A_ref_*, *B_ref1_* and *C_ref1_* in four conditions (*A_ref_* was used in two of these) pseudo-randomly interleaved to provide four independent psychometric functions of 100 trials from each 400-trial run. Participants were encouraged to take breaks around every 100–150 trials. The distance of the comparison square presented in the second interval was chosen using a standard staircase procedure based on Cornsweet's method [15], [16], [39], but modified so that the comparison square was never shown behind the back wall. The proportion of trials on which the comparison was judged as ‘farther away’ was plotted as a function of vergence angle (rather than target distance) by assuming an interocular separation, or lateral translation of the observer, of 6.5 cm. The psychometric function was fitted with a cumulative Gaussian by probit [40]. Figures 2, 3 and 5 plot the point of subjective equality, PSE, i.e. the 50% point, and error bars in Figure S1 show the standard error of the PSE (s.e.m.) derived from this fit.

## Supporting Information

Figure S1 **Using pairs of references to find an interpolated point of subjective equality for square D.** (A) **The distances of the reference squares B and C.** Figure 3 in the paper shows two reference distances and the ‘ideal’ reference distance for square C. The ‘ideal’ reference distances for square C are shown here for all conditions (blue circles), i.e. the PSEs of square C compared with reference square A. In practice, two different reference distances, *C_ref1_* and *C_ref2_*, (shown by crosses), were chosen against which the distance of the square D was judged. Red squares and crosses show, similarly, the PSE of square B compared to A and the reference distances *B_ref1_* and *B_ref2_*. Data are shown for five participants when reference square A was at 0.75 m and 1.5 m and for three participants when reference square A was at 3 m. (B) **Points of subjective equality (PSEs) before interpolation.** Figure 3 also shows an example of an interpolated PSE at which the square D would be perceived to be at the same distance as the ‘ideal’ reference distance for square C. Here we show the interpolated PSEs for all conditions and the original PSEs from which they were derived (open symbols). The PSE of the square D was measured relative to two reference squares, *B_ref1_* and *B_ref2_* (PSEs shown as two red open symbols), and relative to two other reference squares *C_ref1_* and *C_ref2_* (blue). Error bars show the s.e.m. from the probit fit, although in most cases these are smaller than the symbols.
(EPS)

Figure S2 **Interpolation of PSEs.** (A) The PSEs for square D are plotted against the distance of the reference square for two participants S1 and S2. In the top left panel, the red circles show the PSEs of the comparison square D for two reference distances of square B (*D_B1_* and *D_B2_*). These were the data used to generate values shown in Figures S1A and S1B and used to derive the interpolated data in Figure 4. The vertical line shows the distance at which a square B was perceived to be at the same distance as the reference square A (PSE *B_A_*). The solid horizontal line shows the interpolated PSE for the square D assuming that the reference square B was at distance *B_A_* (by interpolating between PSE *D_B1_* and *D_B2_*). The open symbols show additional data taken at other distances of the reference square. Using a linear regression through all five data points gives rise to a very similar estimate (horizontal dashed line) to that obtained using only two points (solid line). The lower panels show similar data, but for reference square C rather than B. Standard errors (s.e.m.) of the matched vergence angle, derived from the probit fit of the psychometric function, were in the order of 1–3 arc minutes.
(EPS)

Figure S3 **Re-plot of** **Figure 5** **without using normalised ranges.** (A) This shows the PSEs *D_B1_*, *D_B2_* (red) and *D_C1_*, *D_C2_* (blue), relative to an unbiased estimate of *D* (*y* = 0, see text) plotted against the vergence angle of the reference square used in each case (i.e. *B_ref1_*, *B_ref2_*, *C_ref1_* or *C_ref2_*). The latter are shown relative to the ‘ideal’ reference value (*x* = 0) for that condition (see text). All values are shown as vergence angles. This is the same as Figure 5 except that the axes have not been normalised by *σ_x_* and *σ_y_* (see text). As in Figure 5, the blue crosses and red plusses show the interpolated values, and *D_B_* and *D_C_* plotted at the ‘ideal’ reference value (*x* = 0). Different symbols show data for different participants.
(EPS)

Procedures S1 **Experimental procedures.**
(PDF)

Procedures S2 **Analysis.**
(PDF)
