# Supplementary material for: A Demonstration of ‘Broken’ Visual Space
Source: PLoS One. 2012 Mar 29;7(3):e33782. doi: 10.1371/journal.pone.0033782 (PMC3315588; doi:10.1371/journal.pone.0033782)
Supplement: Procedures S1 — Experimental procedures. (PDF) [file pone.0033782.s004.pdf]

Experimental Procedures

Target location

During one interval of a trial, the location of the target (either reference or comparison square) remained fixed with respect to the room. Although the participant was continually moving during a trial, 'target distance' was defined relative to a single point in the room, T_0, as follows. T_0 was the location of participant's cyclopean point at the moment they entered a small 'trigger' zone (a tall invisible cylinder of 10 cm radius positioned 0.25 m in depth into the room and 0.43 m from the right hand wall in the 'small' room). The depth dimension was in the direction perpendicular to the back wall and distance was in depth measured relative to T_0. The lateral position of the targets was as follows: targets A and B were positioned to the left of T_0 at an eccentricity of 16, 8, and 3 deg for the three different viewing distances shown in Figure 4 of 0.75, 1.5 or 3 m respectively (i.e. values of A_{ref}). Within a run of 400 trials, the eccentricity of the targets A and B was constant. The same distances and eccentricities applied to targets B and D except that they were positioned on the right. The participant was instructed to move from side to side in order to gain an impression of the distance to the square. The square was visible only when the participant's head (cyclopean point) was within a viewing zone of 1.25 m wide and 0.5 m deep with respect to T_0. So, the participant had to pass through a small trigger zone to initiate the display of the square and then keep within a larger viewing zone for the square to remain visible. However, a table in front of the participant provided a physical restriction. The participant was asked to keep close to the table during experiments so that in practice the range of forward and backward movement with respect to T_0 was small (approximately 24 cm (Svarverud *et al*, 2010)).

Centre of expansion

On trials where the room expanded, the expansion occurred in all directions so that the room became wider, deeper and taller than the 'small' room in the first interval. The textures on the wall, ceiling and floor also expanded with the room, such there were the same number of bricks/tiles in both the 'small' and 'large' rooms. The locus of expansion was a dynamically moving point that coincided with the participant's instantaneous cyclopean optic centre, half way between their eyes. Thus, even as the participant moved during the expansion, there was no visual information about the expansion from the cyclopean point. Despite the fact that participants viewed the scene in stereo (and, hence, from locations slightly displaced from the cyclopean optic centre) the gradual expansion of the room helped to ensure that participants never noticed the room changing size.

Timing of trial intervals

There were spatial constraints that imposed limitations on the timing of the trial intervals. The first interval lasted for at least 2 s, ending when the participant's head returned to a location within the trigger zone (see above). The inter-stimulus interval (ISI) was at least 1 s, likewise ending when the participant re-entered the trigger zone. In the ISI, on those trials in which the room expanded, the room expanded at a linear rate for 1 s until the room was four times its original size. The second interval lasted exactly 2 s. This procedure ensured that the comparison square was presented at the appropriate location and with the same angular size relative to the reference square. Participants were allowed to move with an amplitude and frequency that they found comfortable. They developed

a rhythm when moving from side to side throughout the trials (Svarverud *et al*, 2010), where oscillations were about 0.4-0.5 Hz) so, during the experiments, the intervals were almost always close to the minimum periods.
