# Supplementary material for: A Demonstration of ‘Broken’ Visual Space
Source: PLoS One. 2012 Mar 29;7(3):e33782. doi: 10.1371/journal.pone.0033782 (PMC3315588; doi:10.1371/journal.pone.0033782)
Supplement: Procedures S2 — Analysis. (PDF) [file pone.0033782.s005.pdf]

Analysis

Raw PSEs

Figure 4 in the paper shows points of subjective equality (PSEs) derived from two measured PSEs by interpolation or extrapolation. Figure S1B plots the measured PSEs (open symbols) used to calculate the derived PSEs (solid symbols) that are shown in Figure 4. As explained in the paper, the measured PSEs are the points of subjective equality for square D when compared to reference squares at distances B_{ref1}, B_{ref2}, C_{ref1} and C_{ref2}. Figure S1A shows these reference distances (crosses). It can be seen that in most instances the two reference distances used for each condition span the 'ideal' reference distance (solid symbols), i.e. the PSE B_A or C_A at which square B or C is perceived to be at the same distance as square A. Where this is not the case, the 'ideal' reference distance is usually very close to one of the two actual reference distances.

Error bars

In Figure 4, error bars are plotted for four of the PSEs shown. We explain here how these were derived. Each PSE plotted in Figure 4 was obtained by interpolating between two PSEs that were gathered using reference squares placed at two different reference distances (see Figures S1A and S1B). In order to gain an estimate of the variability of the interpolated value that might be expected across repeated runs of the experiment, we re-ran the experiment with two participants (S1 and S2) using a wider range of reference distances for squares B and C (B_{ref} and C_{ref}) across different runs. As before, only one value of B_{ref} and C_{ref} was used in each run of 400 trials. The range of reference distances can be seen in Figure S2 and, as expected, these give rise to a wider range of PSEs.

The line of best fit can be used to estimate the PSE when the reference was at the 'ideal' distance, (B_A or C_A, shown by the black vertical line in Figure S2). This estimate of the PSE is shown by the dashed horizontal line. In three of the cases in Figure S2 this estimate is extremely close to the estimate obtained from linear interpolation using only the two points used in the main experiment (solid symbols and solid horizontal line). In the other case (participant S2 for reference B), the difference is greater. We used the variability of the PSEs about the line of best fit to provide a bootstrap estimate of the reliability of interpolation using only two reference distances, as follows.

For each data set shown in Figure S2, the five differences between the PSE and the regression line were sampled randomly (with replacement) and new points generated at the two reference distances used in the experiment. For each new pair, a PSE was calculated by interpolation in the same way as it was for the original data. For a large number of repeats, the standard deviation of the interpolated PSEs asymptotes. This was the value used to plot the error bars in Figure 4. Extrapolation, when it occurred, was usually minimal. It was used in five out of twenty-six cases (see Figure S1B), but in three of these cases one of the two references fell within one standard error of the PSE that indicated the 'ideal' reference distance (i.e. one s.e.m. of B_A or C_A) .

Direct comparison of squares A and D

Under normal, unconstrained viewing conditions, it is possible to look freely between objects and to make many pairwise comparisons between the distances of objects, not just the restricted set that we examined in our experiment. For two participants, we measured the perceived distance of square D relative to reference square A when compared

directly, without any intervening distance judgement. The PSEs in this case are shown by the grey triangles in Figure 4. In this condition, the room expanded between intervals and the location of the square moved from the centre to the side of the room. For both participants, the point of subjective equality was in between that obtained for the routes via square B or C, as one might expect.

Data normalisation for Figure 5

The values plotted on the abscissa of Figure 5 are defined as follows:

$$x = (x_1 - x_0)/\sigma_x \tag{1}$$

where x_1 is the reference value used (e.g. B_{ref1}) and x_0 is the 'ideal' reference value (i.e. the PSE of the square in that location relative to the reference square A, e.g. PSE B_A). σ_x is the standard deviation of the fitted psychometric function when the distance of the square in that location was judged relative to the reference square A (e.g. the grey psychometric functions in Rows I or III of Figure 2).

The values plotted on the ordinate of Figure 5 are:

$$y = (y_1 - y_0)/\sigma_y \tag{2}$$

where y_1 is the measured PSE of square D (e.g. PSE D_B) and y_0 is the 'expected' PSE assuming the reference (B or C) was at the 'ideal' location. We took the mean of the interpolated PSEs D_B and D_C as an unbiased estimate of y_0. We computed σ_y as:

$$\sigma_y = \sqrt{\sigma_{y1}^2 + \sigma_{y2}^2 + \sigma_{y3}^2 + \sigma_{y4}^2} \tag{3}$$

where σ_{y1} to σ_{y4} are the standard deviations of the fitted psychometric functions yielding PSE D_{B1}, D_{B2}, D_{C1} and D_{C2} (and the latter two could be, for example, the two blue psychometric functions in Figure 3). Figure S3 below is the same as Figure 5 in the paper except that the x and y values have not been divided by σ_x and σ_y respectively.
